# Intracranial dissemination of *Klebsiella pneumoniae* originating from pulmonary infection: a case report

**DOI:** 10.1186/s13256-024-04653-6

**Published:** 2024-07-14

**Authors:** Ghazaleh Jamalipoursufi, Ali Hajihashemi, Shokouh Sadeghizade, Mahsa Geravandi

**Affiliations:** Department of Radiology, Isfahan University of Medical Sciences, Isfahan, Iran

**Keywords:** *Klebsiella pneumoniae*, Metastatic brain abscesses, Brain abscesses, Pleural abscess

## Abstract

**Background:**

Metastatic brain abscesses caused by *Klebsiella pneumoniae* are extremely rare but life-threatening conditions. To depict a unique case of the middle-aged hypertensive man with an unusual presentation of metastatic brain abscesses originating from a pleural abscess caused by *Klebsiella pneumoniae* and subsequently leading to loss of consciousness (LOC).

**Case report:**

A 52-year-old Iranian man with a history of hypertension presented to the emergency department with a five-day history of worsening cough, high-grade fever, shortness of breath, chest pain, fatigue, and a productive cough. Laboratory tests revealed leukocytosis, elevated C-reactive protein, and respiratory alkalosis. A chest computed tomography scan confirmed pneumonia, and a brain scan revealed multiple hypodense lesions. Despite antibiotic therapy, the patient's condition worsened, leading to confusion, disorientation, and loss of consciousness. Magnetic resonance imaging revealed multiple ring-enhancing lesions, suggesting an abscess formation. Bronchial washings and BAL samples confirmed a lower respiratory tract infection. Cultures from the bronchial washings grew *Klebsiella pneumoniae*.

**Conclusions:**

Metastatic brain abscesses caused by *Klebsiella pneumoniae* are exceedingly rare but life-threatening conditions. Timely diagnosis and effective antimicrobial treatment are critical for patient outcomes. This case underscores the significance of recognizing atypical presentations of bacterial infections, as early detection and appropriate management can significantly impact patient outcomes.

## Introduction

Brain abscesses are rare, life-threatening conditions characterized by pus collection within the cerebral parenchyma [1]. The annual incidence of brain abscesses globally ranges from 1500 to 2500 cases, with mortality rates ranging from 5 to 32% [2, 3]. Brain abscesses can appear with various clinical manifestations. The well-known triad of brain abscess is seen in only 20% of patients which includes fever, headache, and focal neurologic deficits(FND). patients with these abscess may present with only progressive deterioration in cognition or behavior, without fever or FNDs [2]. Brain abscesses are caused by infections from distant sites including but not limited to sinusitis, subacute or chronic otitis media, and mastoiditis, which constitute a predominant mode of transmission. Additional contributing elements encompass trauma, surgery, odontogenic infections, hematogenous dissemination, and sporadic cases of cryptogenic origin [4–6]. Metastatic brain abscesses, particularly those involving *Klebsiella pneumoniae*, are rare and challenging. *Klebsiella pneumoniae* is a type of encapsulated gram-negative bacterium that is infrequently identified as the causative pathogen for brain abscesses. While sporadic reports exist on brain abscesses caused by primary *Klebsiella pneumoniae* infections, instances of metastatic brain abscess due to this pathogen are exceedingly uncommon and require further investigation [4, 7, 8].

This case report reveals the complexities of diagnosing, managing, and addressing these rare cases, emphasizing the need for further scrutiny and potential patient care implications.

### Case presentation

A 52-year-old Iranian man with a history of hypertension presented to the emergency department with a five-day history of worsening cough, high-grade fever, and shortness of breath. He also reported chest pain, fatigue, and a productive cough with yellow sputum. He denied any recent travel or exposure to sick contacts. His medical history was significant for well-controlled hypertension and he did not have diabetes mellitus. No history of tobacco smoking, alcohol, or substance abuse was noted.

On physical examination, He was conscious with a Glasgow Coma Scale (GCS) score of 15(E4V5M6). he appeared acutely ill, with tachypnea, and increased work of breathing, and pulmonary auscultation revealed decreased breath sounds and coarse crackles over both lung fields. Upon admission, the following vital signs were detected; heart rate of 95 beats/min, respiratory rate of 24 breaths/min, blood pressure of 110/80 mmHg, temperature of 39.5°C, and oxygen saturation of 89% on room air. The cardiovascular examination was unremarkable, along with other system examinations.

Laboratory investigations showed leukocytosis with a white blood cell count of 15,500/μL (reference range: 4500–11,000/μL) and a left shift. The C-reactive protein (CRP) level was markedly elevated at 30 mg/dL (reference range: < 0.5 mg/dL), indicating a significant inflammatory response. Arterial blood gas analysis revealed respiratory alkalosis with partial compensation. The patient's blood glucose level was 110 mg/dL (reference range: 70–140 mg/dL). Other laboratory tests such as antinuclear antibodies, hepatitis, coagulation test, thyroid function, vasculitis screening, Brucella agglutination test, purified protein derivative (PPD) for tuberculous infection, and HIV showed no obvious abnormalities.

The chest computed tomography (CT) with contrast showed consolidation in both lower lobes, which is consistent with a diagnosis of pneumonia. The right costophrenic angle was blunted, indicating pleural effusion. Also, the loculated fluid collection was noticed in the right side posterior pleural space in favor of the pulmonary abscess. (Fig. 2A) The patient was promptly started on empirical antibiotic therapy with intravenous ceftriaxone and supplemental oxygen therapy via nasal cannula after a diagnosis of community-acquired pneumonia was made. Over the next few days, the patient's clinical condition deteriorated despite the administration of antibiotics. His mental status deteriorated, including confusion, disorientation, and eventually loss of consciousness. The neurological examination disclosed a Glasgow Coma Scale (GCS) score of 6 (E2V2M2), indicating unresponsiveness. Delayed pupillary reflexes and decerebrate posturing were observed.

**Fig. 1:**
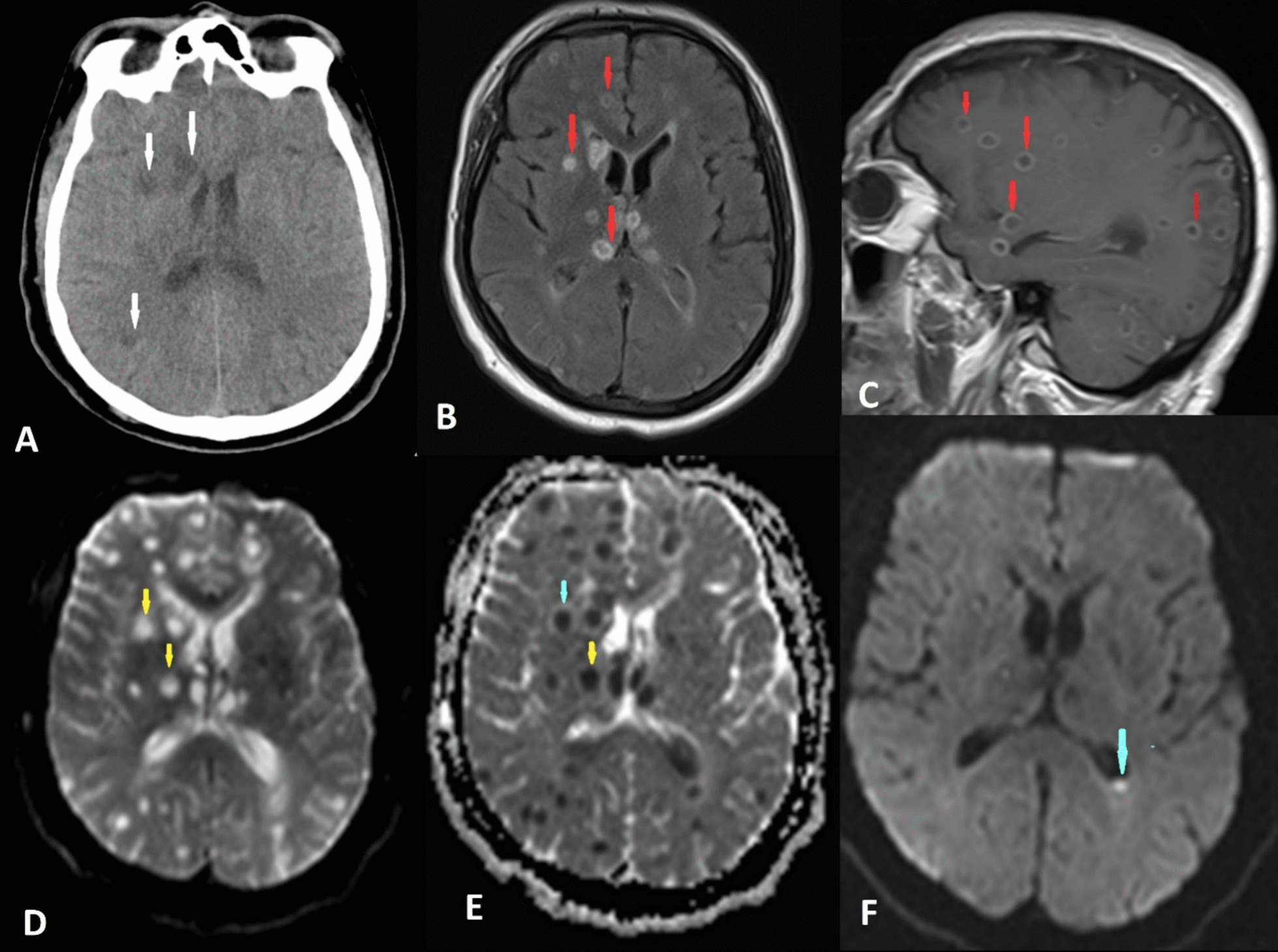
Brain computed tomography scan and magnetic resonance imaging at admission. **A** The brain computed tomography scan showed multiple supra- and infratentorial intra-axial hypodense lesions in both cerebral hemispheres and basal ganglia (white arrows). Axial FLAIR sequence, sagittal FLAIR sequence (**B**, **C**), axial T2W (**D**), and ADC map (**E**): Multiple distinct outlined intra-axial ring-enhancing lesions distributed within the supra- and infratentorial cerebral parenchyma, alongside the periventricular region (red and yellow arrows in **B**, **C** and **D**). Notably, the central portions of these lesions exhibit noticeable diffusion restriction, and there is mild peripheral edema surrounding the lesions (yellow and blue arrows in **E**). Brain MRI after treatment (**F**): Both the quantity and the extent of lesion restriction had visibly diminished. Furthermore, ventriculitis was confined to the left occipital horn (blue arrow in **F**)

A computed tomography (CT) scan of the brain was performed immediately. It showed multiple supra- and infratentorial intra-axial hypodense lesions in both cerebral hemispheres, which were probably caused by an infectious process and less likely metastatic lesions. (Fig. 1A) For further evaluation, magnetic resonance imaging (MRI) of the brain was conducted. The MRI revealed multiple well-defined, intra-axial ring-enhancing lesions, measuring approximately 10mm in diameter in the supra- and infratentorial cerebral parenchyma, as well as in the periventricular region. The center of the lesions showed prominent diffusion restriction and mildly similar peripheral edema around the lesions was also noted. The ring enhancement was indicative of a necrotic center surrounded by an enhancing rim, suggestive of abscess formation. Additionally, intraventricular material exhibited diffusion restriction, indicating the presence of ventriculitis. Increased subarachnoid spaces around optic nerves were suggestive of increased intracranial pressure (ICP). (Fig. 1B–E) The common cerebral spinal fluid (CSF) results as CSF cultures, acid-fast dyeing, and India ink staining were negative.

Antibiotic therapy was instituted empirically with intravenous ceftriaxone and metronidazole to cover both the suspected pneumonia and potential brain abscess. Blood cultures were obtained on admission and showed no bacterial growth. A bronchoscopy was performed to obtain a lower respiratory tract sample for culture. Bronchial washings and bronchoalveolar lavage (BAL) samples were collected and sent for microbiological analysis. The bronchoscopy revealed purulent secretions in the bronchial tree, confirming the presence of a lower respiratory tract infection. Cultures from the bronchial washings grew *Klebsiella pneumoniae*. The results of the antibiotic susceptibility test were amoxicillin/clavulanate, ceftazidime, cefazolin, imipenem, and meropenem.

Based on the clinical presentation, laboratory findings, and imaging results, the initial diagnosis pointed toward metastatic brain abscesses caused by *Klebsiella pneumoniae*. To enhance the range of effective antimicrobial treatments, meropenem was chosen as a substitute for ceftriaxone and metronidazole. Additionally, to alleviate intracranial pressure, mannitol 20% was administered. The patient was transferred to the intensive care unit (ICU) for continued antibiotic therapy and close monitoring. Moreover, a contrast-enhanced abdominopelvic CT scan was conducted, which revealed no abnormal masses or lesions within the solid organs. Otherwise, the findings from the study were unremarkable.

For the purpose of tracking treatment efficacy, serial MRI images of the brain were obtained. The outcomes revealed that certain lesions exhibited slight diffusion restriction in diffusion-weighted imaging (DWI). Notably, both the quantity and the extent of lesion restriction had visibly diminished. Furthermore, ventriculitis was confined to the left occipital horn, signifying a progressive reduction in brain lesions and localized ventriculitis regression (Fig. 1F).

**Fig. 2:**
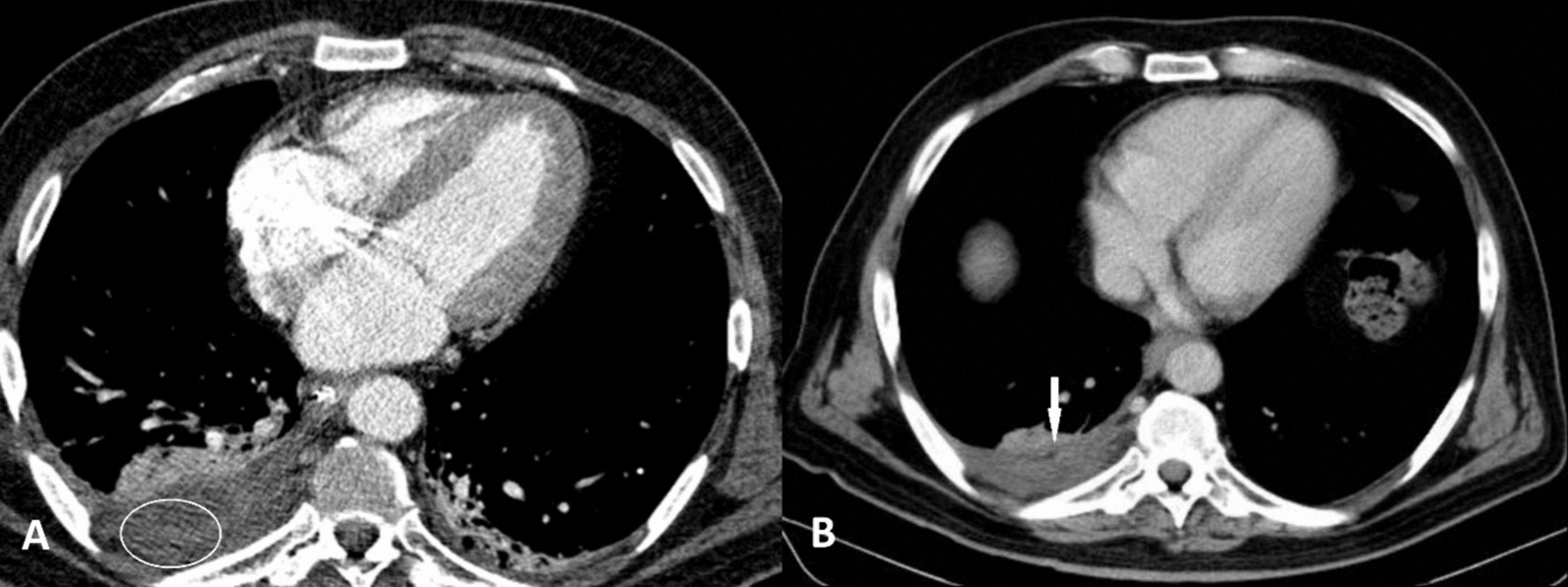
Chest computed tomography scan with IV contrast. **A** at the time of admission, showed a loculated fluid collection with mild wall enhancement in the right side posterior pleural space in favor of the pulmonary abscess (white oval). **B** After treatment, a gradual decrease in the size and extent of the abscesses was observed (arrow)

Sequential chest CT scans were executed to observe the advancement of the pulmonary abscesses. The findings unveiled a gradual diminishment in the size and extent of the abscesses (Fig. 2B).

Following an 8-week therapeutic course, the patient was discharged with a stable condition, showing no signs of neurological deficits. A subsequent cranial MRI conducted 24 months after the treatment exhibited a significant reduction in the brain abscesses. The most recent outpatient clinical follow-up assessment confirmed the patient's continued physical and neurological stability.

## Discussion

In this case report we present a unique case of a 52 years old man who was admitted to our department due to an episode of *Klebsiella pneumoniae* pneumonia. Following several days of hospitalization, the patient exhibited distinct neurological manifestations, prompting an extended diagnostic assessment. Subsequent evaluation definitively established the presence of metastatic brain abscesses attributable to *Klebsiella pneumoniae.*

The infection's access to the brain occurs through four distinct pathways. The predominant route, accounting for 45–50% of cases, involves contiguous suppurative focus. Alternative pathways encompass transmission through trauma, hematogenous dissemination, and cryptogenic origin [7, 8].

The hematogenous source of brain abscesses can arise from various organs, including the heart, lungs, skin, and intraabdominal and pelvic infections [9].

Abscess formation through the hematogenous route can occur in a multifocal manner, as observed in our presented case [5].

Given the unique attributes of our case, its rarity, and the distinctive source of the abscess, as well as its metastatic nature, it holds significance for reporting. Furthermore, the infrequent occurrence of pneumonia caused by this particular pathogen within this context adds another layer of importance to its documentation.

In a recent case report, an unusual occurrence was noted in an adult patient presenting with a *Klebsiella pneumonia* brain abscess, where no identifiable source of Klebsiella infection was found [10, 11]. In contrast, our case involved a lung infection as the suspected source.

A case report showed the importance and lethality of Klebsiella brain abscesses and emphasized its prompt and timely treatment. A healthy middle-aged man presented with progressive altered mental status and behavioral change and was diagnosed Klebsiella induced brain abscess. Due to spillage of abscess into the cerebral ventricles during surgery, the patient's condition worsened rapidly and eventually led to the patient's death [7]. This study is different from our study because in our case, despite the findings of ventriculitis in imaging, timely treatment prevented the death of the patient.

Additionally, another article highlighted a rare case of a patient with a history of chronic lymphocytic leukemia who developed a *Klebsiella pneumonia* brain abscess [12]. It is worth noting that our case differs as the patient does not have any underlying immunodeficiency.

In a recent case report, an 81-year-old woman was documented to have a *Klebsiella pneumonia* brain abscess without any neurological clinical presentation [13]. This finding stands in contrast to our own case, where neurological manifestations were observed.

## Conclusion

This case report underscores the importance of considering metastatic brain abscess as a potential complication in patients with *Klebsiella pneumonia*, particularly when neurological symptoms arise. It emphasizes the need for early recognition, prompt diagnosis, and appropriate management strategies. In some cases, a multidisciplinary approach combining various treatment modalities may be necessary to optimize patient outcomes.

## Data Availability

All data and materials are available from the corresponding author upon request.
